# Complete mitochondrial genome of the Mongolia pheasant, *Phasianus colchicus kiangsuensis* (Galliformes, Phasianidae, Phasianus)

**DOI:** 10.1080/23802359.2016.1174089

**Published:** 2016-06-20

**Authors:** Qiong Wu, Yang Li, Chao Song, Haoran Ning, Xiumei Xing

**Affiliations:** a Institute of Special Wild Economic Animals and Plants Science, Chinese Academy Agricultural Science, Changchun, Jilin Province, PR China;; b State Key Laboratory of Special Economic Animal Molecular Biology, Changchun, Jilin Province, PR China

**Keywords:** Mitochondrial genome, Mongolia pheasant, *Phasianus colchicus kiangsuensis*, phylogenetic analysis

## Abstract

The pheasant (*Phasianus colchicus*) *is* raised by commercial farms in most parts of China because of special fleshy flavour. In the study, complete mitochondrial genome of the Mongolia pheasant was sequenced by polymerase chain reaction (PCR) as well as the primer walking sequence method. The entire mitochondrial genome of Mongolia pheasant was 16,673 bp in length, gene composition and arrangement conformed to most bird, which contained the typical structure of 22 tRNAs, 2 rRNAs, 13 protein-coding genes and a non-coding region. The phylogenetic tree of 20 *Phasianidaes* showed that Mongolia pheasant had close relationship to ring-necked pheasant. Our complete mitochondrial genome sequence will be useful phylogenetics, and be available as basic data for the breeding and genetics.

Today, Mongolia pheasant have been farmed widely in China. In this study, complete mitochondrial genome of Mongolia pheasant (*Phasianus colchicus kiangsuensis*) was sequenced.

The Mongolia pheasant was randomly selected from rare birds breeding farm in Shanghai. Venous blood was collected before slaughter, and coagulation was prevented with citrate. Genomic DNA was extracted from thawed blood using the saturated salt method and stored at −20 °C. Polymerase chain reaction amplification, sequencing and sequence analysis were completed according to He et al. (2009). The mitochondrial genome sequence has been submitted to Genbank (accession no. KP637175).

The mitochondrial genome of Mongolia pheasant is 16,673 bp in length. It contains 22 tRNA genes, two rRNA genes, 13 protein-coding genes and a non-coding region, most genes are encoded on the H-strand, which are similar to the typical mitochondrial genomes of vertebrate (Boore 1999). Within the Mongolia pheasant mitochondrial genome sequence, the nucleotide composition is 30.63% A, 30.88% C, 25.26% T and 13.22% G. It contains 46 bp spacer sequence (16 sites), and 39 bp overlapping sequence (eight sites). The non-coding region is 1149 bp in length, which is located between the tRNA*^Phe^* and the tRNA*^Glu^*, accounting for 6.88% of all genes.

All protein-coding genes of mitochondrial genome were identified by comparing with published sequences of other pheasants. The total lengths of the 13 protein-coding genes are 12,303 bp. Within the 13 protein-coding genes, apart from ND6 gene encoded on the L-strand, the remaining protein-coding genes are encoded on the H-strand. All protein-coding genes initiate with codon ATG except *COI*. The stop codon TAA is common, *COI* terminate with AGG, *CYTB* terminate with TAG, *ND2, COIII, ND4* and *ND6* appear to end in incomplete stop codon T. The incomplete stop codon T is presumably completed as TAA by post-transcriptional polyadenylation (Boore 1999, 2004).

It contains 22 tRNA genes and 2 rRNA genes. The sizes of the 22 tRNA genes range from 58 to 78 bp. The 12S rRNA and 16S rRNA are located between the tRNA*^Phe^* and the tRNA*^Leu (UUR)^*, and separated by the tRNA*^Val^*. The lengths of the 12S rRNA and 16S rRNA are 965 and 1621 bp, respectively.

The phylogenetic analysis of Mongolia pheasant with other *Phasianidaes* were performed used the MEGA 5.0 software (MEGA Inc., Englewood, NJ). The phylogenetic tree showed that Mongolia pheasant had close relationship to ring-necked pheasant (Figure 1).

**Figure 1.**
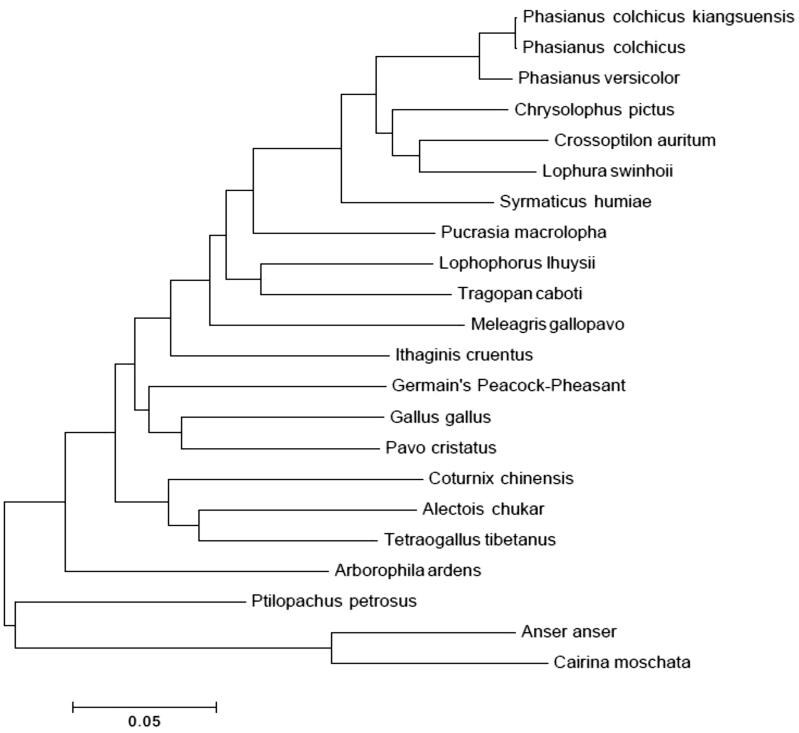
The phylogenetic tree among 20 *Phasianidaes*.

In summary, the Mongolia pheasant mitochondrial genome sequence can provide the resource for the breeding and genetics. Furthermore, classification of the pheasant has less information, and lack of consensus conclusion, the complete mitochondrial genome sequence will provide an additional tool for understanding the relationship of the pheasant with other rare birds.
